# Endodontic and Periodontal Treatment of a Two‐Rooted Maxillary Lateral Incisor With a Type III Palatoradicular Groove: A Case Report With 2‐Year Follow‐Up

**DOI:** 10.1155/crid/2543929

**Published:** 2026-02-20

**Authors:** Katsuhiro Takeda, Tomoya Naruse, Yohei Takahashi, Reina Kawai, Kimiaki Yuhi, Hideki Shiba

**Affiliations:** ^1^ Department of Biological Endodontics, Graduate School of Biomedical and Health Sciences, Hiroshima University, Hiroshima, Japan, hiroshima-u.ac.jp

**Keywords:** endodontic–periodontal lesion, endodontics, palatoradicular groove, periodontics

## Abstract

**Introduction:**

A palatoradicular groove (PRG) is a developmental anomaly that usually starts from the central fossa, crosses the cingulum, and extends apically onto the root surfaces at varying degrees. It can lead to both periodontal and endodontic issues in clinical practice. Therefore, early diagnosis and treatment of PRGs are crucial for improving patients′ prognosis.

**Case Report:**

A 29‐year‐old woman complained of discomfort around the upper left lateral incisor. Three‐dimensional constructed CBCT images clearly showed that #22 had a supernumerary root. Tooth #22 was diagnosed as having an endodontic–periodontal lesion caused by a Type III PRG and an additional root. This case report presents a case of endodontic–periodontal lesions caused by a PRG of #22 with two roots, in which root canal treatment and surgical periodontal procedures provided good outcomes.

**Conclusion:**

In‐depth knowledge of anatomical variations of the morphology of the root canal system of PRG is necessary for accurate diagnosis and successful treatment. CBCT showed accurate anatomical details, which were helpful for planning the treatment of the tooth with a PRG.

## 1. Introduction

A palatoradicular groove (PRG) is a developmental anomaly that usually starts from the central fossa, crosses the cingulum, and extends apically onto the root surfaces at varying degrees [1, 2]. It is predominantly found on the palatal aspect of maxillary central and lateral incisors [3]. The incidence of PRGs is reported to be between 0.9% and 4.6% [2, 4, 5] in extracted maxillary incisors and from 2.8% to 18.1% [1, 6] in clinical studies. The variation in incidence rates reported in these papers may be due to study design, race, and region of the participants, sample size, and diagnostic criteria.

Gu categorized the types of prominent PRGs into three categories (Type I, Type II, and Type III) based on the depth and length of the groove, using extracted teeth that were scanned with micro‐CT imaging [7]. A Type I PRG is characterized as short and shallow, not extending beyond the coronal third of the root. In Type II, the groove is longer and extends a significant distance along the root, although it still does not reach beyond the coronal third. A Type III PRG has the most complex anatomy, with a long and deep invagination that goes beyond the coronal third of the root. This complexity indicates a more intricate root canal system. In a Type III PRG, a C‐shaped root canal, an invagination root canal, and an additional root with a secondary root canal are observed.

A PRG requires clinical attention because it is associated with the development of an inflammatory process in periodontal tissues [8]. A PRG becomes a retention factor for dental plaque and dental calculus and contributes to apical migration of dental biofilms and formation of a localized periodontal pocket along the grooves in susceptible hosts [2].

As the destruction of periodontal tissue progresses, bacteria invade the root canal via the accessory canals and apical foramen [9]. This then triggers endodontic–periodontal disease [10, 11]. Teeth with a PRG have a worse prognosis, depending on factors such as the depth and length of the PRG, the extent of periodontal tissue loss, and the anatomical morphology of the infected root canal system [12]. A detailed examination and comprehensive understanding of PRGs, including cone‐beam computed tomography (CBCT) imaging, are essential for clinical success [10]. This case report presents a rare combination of a Type III PRG and a supernumerary root in a maxillary lateral incisor. It highlights how preoperative three‐dimensional morphological analysis using CBCT and its direct incorporation into treatment planning contributed to successful management of this complex endodontic–periodontal lesion. Furthermore, this report provides a three‐dimensional evaluation of bone regeneration over a 2‐year postoperative period. By addressing complex defect morphology—including penetrating lesions with marginal bone loss and fenestrations—this case offers new practical insights into treatment strategies and prognosis of PRG‐associated lesions with severe periodontal destruction. Therefore, this case report is aimed at describing the diagnosis and successful management of this rare and challenging condition and at discussing its clinical implications for future treatment of PRGs.

To clarify the clinical assumptions of this case, the null hypothesis of the present report was that the endodontic–periodontal lesion in Tooth #22 was not associated with the presence of a Type III PRG or the supernumerary root.

## 2. Case Presentation

### 2.1. Diagnosis and Etiology

The present case report was written according to the CARE guidelines (https://www.care-statement.org). In this sense, data were obtained and used according to ethical requirements.

A 29‐year‐old woman was referred from a private dental clinic to the Department of Endodontics and Operative Dentistry of Hiroshima University Hospital. Her chief complaint was discomfort around the upper left lateral incisor. The referral letter indicated that Tooth #22 had a negative response to the electric pulp test. The letter noted that the condition had not improved even though the tooth had undergone nonsurgical periodontal therapy and root canal treatment. It also noted that calcium hydroxide paste (Vitapex, Neo Dental Chemical Products Co. Ltd., Tokyo, Japan) had been placed as a temporary disinfectant dressing into the root canal. The patient did not have any systemic diseases. A sinus tract was observed in the buccal gingiva around #22 (Figure 1a). The patient showed good oral hygiene, and the gingiva around all teeth except #22 exhibited no signs of severe inflammation. Inflammation was present in the central marginal gingiva on the palatal side of #22, characterized by mild redness and swelling (Figure 1b). Pocket probing depth (PPD) on #22 was 2 mm in all areas except the palatal center, where the PPD was 10 mm. Probing in the palatal center resulted in bleeding and discharge of pus. On the radiovisiography (RVG)/intraoral periapical radiograph (IOPA) at the first visit to our hospital, an opaque image was seen in the root canal (Figure 1c). This was the calcium hydroxide paste (Vitapex, Neo Dental Chemical Products Co. Ltd., Tokyo, Japan) mentioned in the referral letter. In addition, a radiopaque image was observed proximal to the root of #22, which appeared to be a supernumerary root (Figure 1c). CBCT images (3D Accuitomo, J Morita, Tokyo, Japan; analyzed using SYNAPSE VINCENT Version 4.6; FUJIFILM, Tokyo, Japan) of #22 showed an extensive bone defect on the mesial proximal aspect (Figure 2a). The buccal and palatal cortical bone of #22 was partially lost, a so‐called through‐and‐through lesion (Figure 2a). Three‐dimensional reconstruction revealed a supernumerary root located on the mesiopalatal aspect of the main root of #22 (Figure 2b). Tooth #22 was diagnosed as having an endodontic–periodontal lesion caused by a Type III PRG and an additional root.

Figure 1Intraoral photographs and dental radiographs at the first visit. (a) Intraoral photograph at the first visit. #22 has a sinus tract (black arrowhead) at the labial gingiva. (b) Redness and swelling are observed at the palatal gingiva around #22 (∗). (c) Dental radiographic examination shows an opaque image in the root canal. A radiopaque image is seen proximal to the root of #22, which appears to be a supernumerary root (black arrow).

**Figure:**
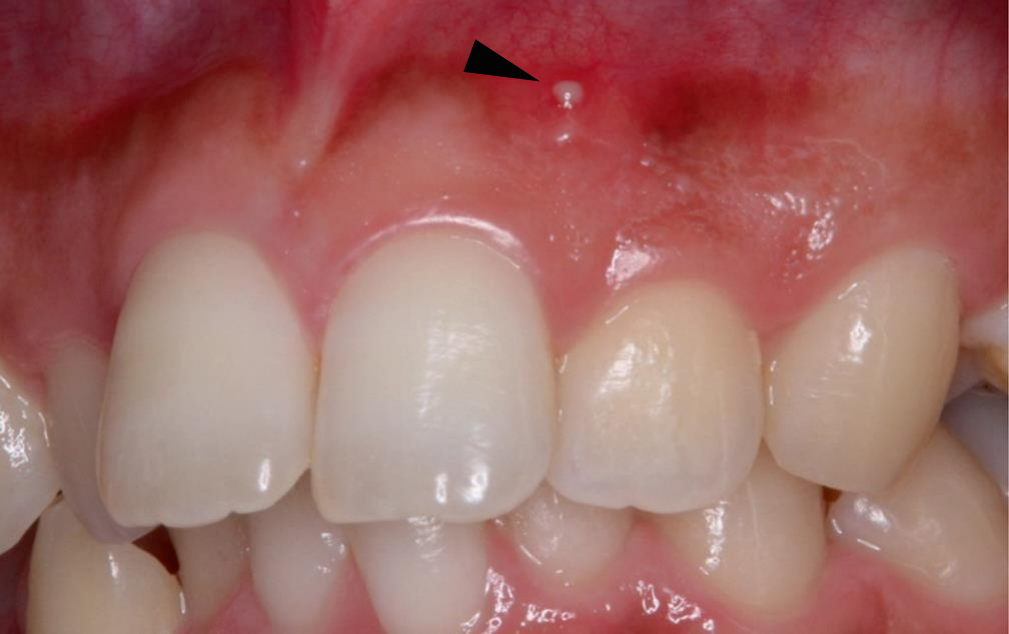
(a)

**Figure:**
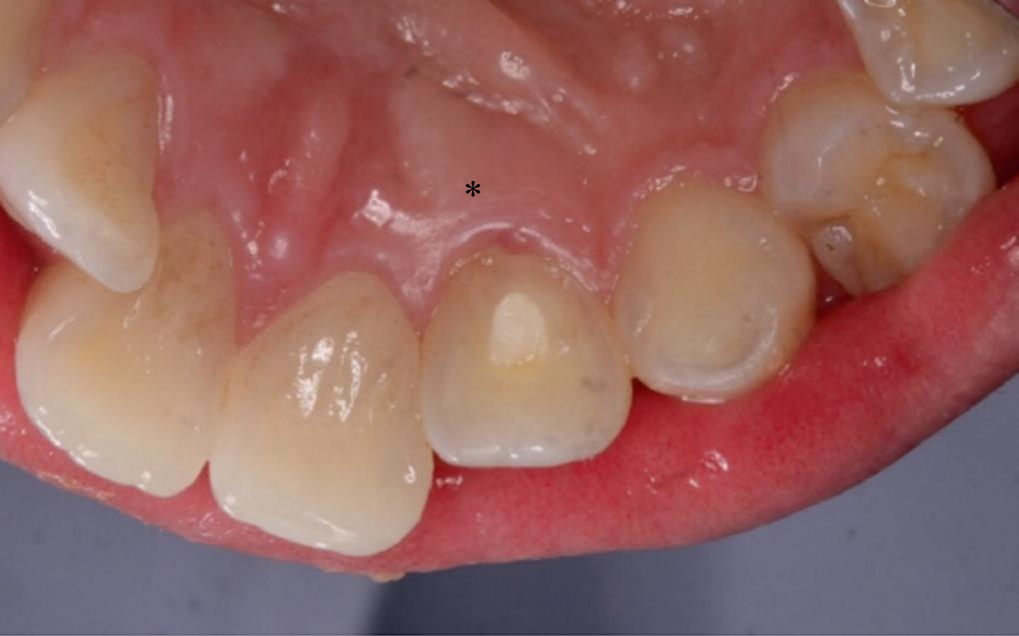
(b)

**Figure:**
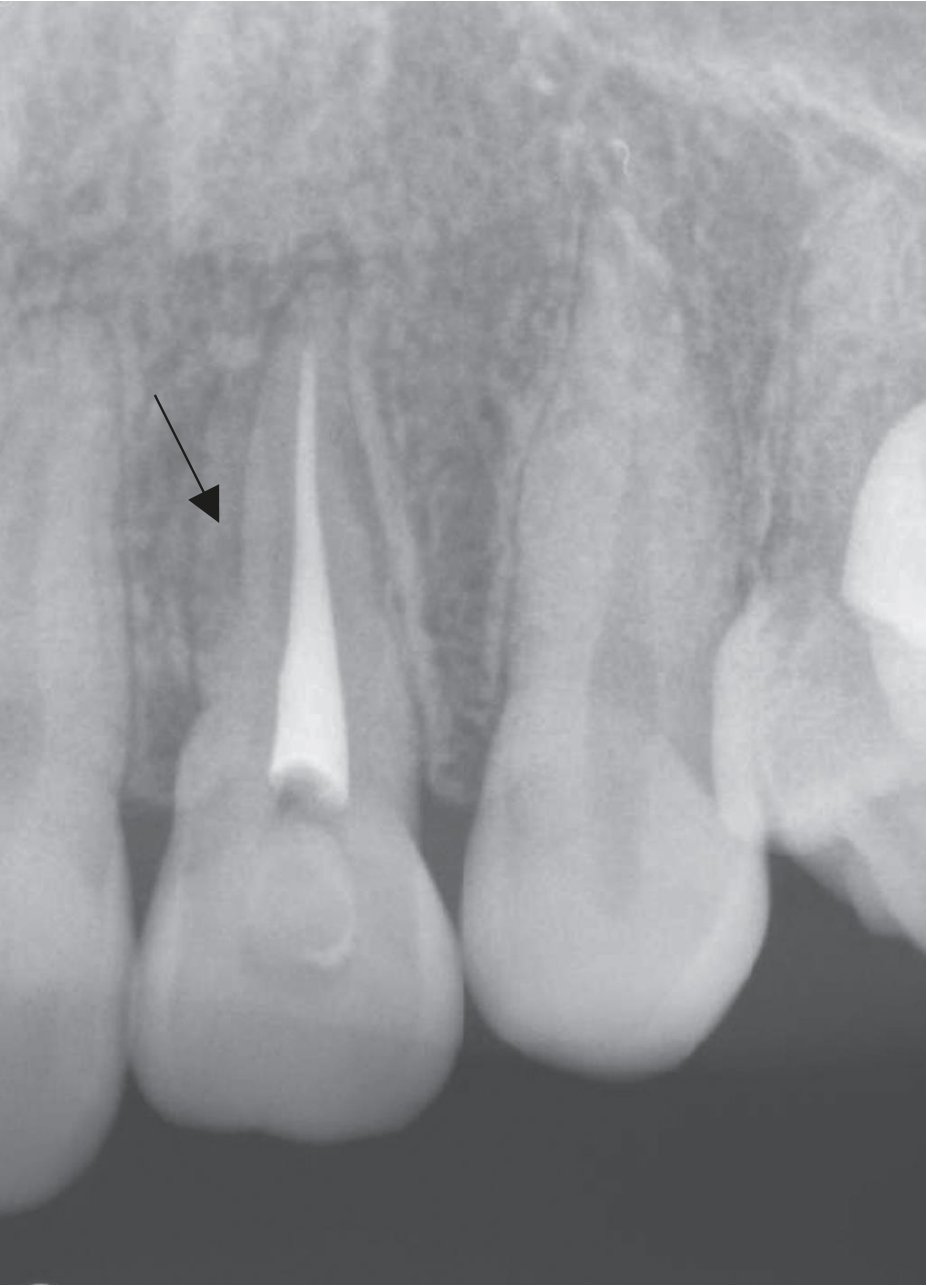
(c)

Figure 2CBCT images of #22 at the first visit. (a) A series of CBCT horizontal images clearly shows the excess root. Arrowhead: labial bony fenestration. (b) A three‐dimensional reconstruction image. White arrow: supernumerary root.

**Figure:**
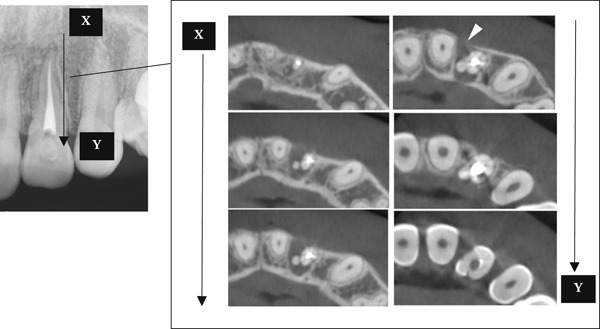
(a)

**Figure:**
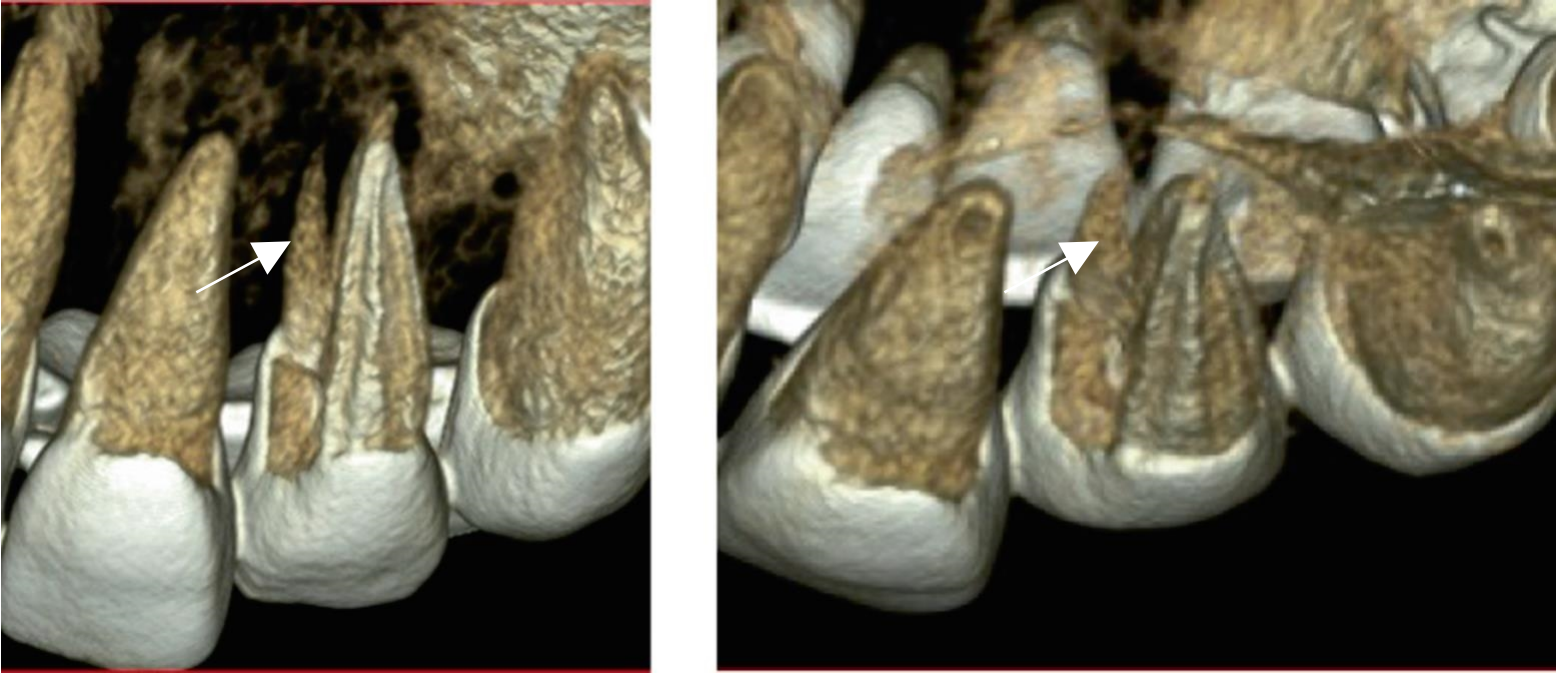
(b)

### 2.2. Treatment Objectives

The objective of treatment was to eliminate the source of infection associated with the Type III palatogingival groove and the supernumerary root, resolve the periodontal defect, preserve Tooth #22, and restore function and comfort. Additional aims included promoting bone healing, eliminating the sinus tract, and achieving long‐term periodontal stability.

### 2.3. Treatment Alternatives

The treatment plan was thoroughly explained to the patient, and her informed consent was obtained. After rubber dam isolation, the temporary sealing was removed, revealing the root canal orifice of the main root. Several attempts were made to locate the orifice of the accessory root canal under magnification but without success. CBCT analysis showed that the accessory root canal originated almost perpendicularly from the main canal. Because of this extremely unfavorable angulation, endodontic instruments could not be inserted into the canal pathway, although no radiographic or clinical signs of canal calcification were observed. Since the canal was not negotiable despite repeated attempts, nonsurgical root canal treatment of the accessory root was deemed impractical. Therefore, root canal treatment for the accessory root was abandoned, and surgical removal of the supernumerary root was selected, leaving the calcium hydroxide paste (Vitapex) in place.

### 2.4. Treatment Progress

During the periodontal surgical phase of the treatment, the surgical area was anesthetized with 3.6 mL of 2% lidocaine with 1:40,000 epinephrine (ORA Injection Dental Cartridge, GC Showayakuhin Co., Tokyo, Japan) after disinfection with povidone‐iodine. A full‐thickness mucoperiosteal flap was created using intrasulcular incisions on both the labial and palatal sides. On the palatal side, after performing curettage to remove granulation tissue from the defect, a teardrop‐shaped defect was revealed (Figure 3a). The PRG emerged from the basal ridge, extending apically and mesially, ultimately terminating in the bifurcation area and giving rise to an extra root (Figure 3a). When the labial flap was reflected, a bony fenestration was observed corresponding to the location of a previously existing sinus tract (Figure 3b). The extra root was resected using an electric handpiece (Ti‐Max X95L, NAKANISHI INC., Tochigi, Japan), and the remaining granulation tissue was curetted out with hand curettes (Gracey Curette No. 1/2 and 7/8, Hu‐Friedy Manufacturing Co., Chicago, Illinois, United States) (Figure 3c). Subsequently, root planing was performed using hand curettes and an ultrasonic scaler (Varios 970, NAKANISHI INC.). A radiculoplasty was carried out using a round bur (Mani) and sealed with composite resin (GRACEFIL Flo, GC Co. Ltd., Tokyo, Japan) (Figure 3d). The flaps were repositioned and sutured with 5‐0 nonresorbable monofilament sutures (SOFTRETCH, GC Co. Ltd.) (Figure 3e). The length of the resected root was approximately 9 mm (Figure 3f). Adherence to the surgical protocol was evaluated through review of clinical records and postoperative follow‐up visits. The patient complied with all scheduled appointments and followed instructions for antimicrobial mouth rinse and wound care. Tolerability was monitored through postoperative clinical assessments and patient‐reported symptoms. Healing was uneventful, and sutures were removed 7 days after the surgery. One month after the surgery, the root canal was filled with gutta‐percha (GUTTA PERCHA POINTS, GC Co. Ltd.) and sealers (NISHIKA CANAL SEALER BG, Nishika Nippon Shika Yakuhin Co., Yamaguchi, Japan) using the lateral condensation technique [13], after which the access cavity was doubly sealed with stopping (Temporary Stopping, GC Co. Ltd.) and glass ionomer cement (Base cement, SHOFU Inc., Kyoto, Japan).

Figure 3Surgical procedure. (a) Surgical opening of the palatoradicular groove. Asterisk: extra root, arrow: palatoradicular groove. (b) The reflected flap on the labial surface shows a bony fenestration corresponding to the location of the previously existing sinus tract. (c) The extra root is resected using an electric handpiece, and the remaining granulation tissue is curetted out with hand curettes. (d) The radiculoplasty is carried out and sealed with composite resin. (e) The flap is replaced and sutured. (f) Resected root.

**Figure:**
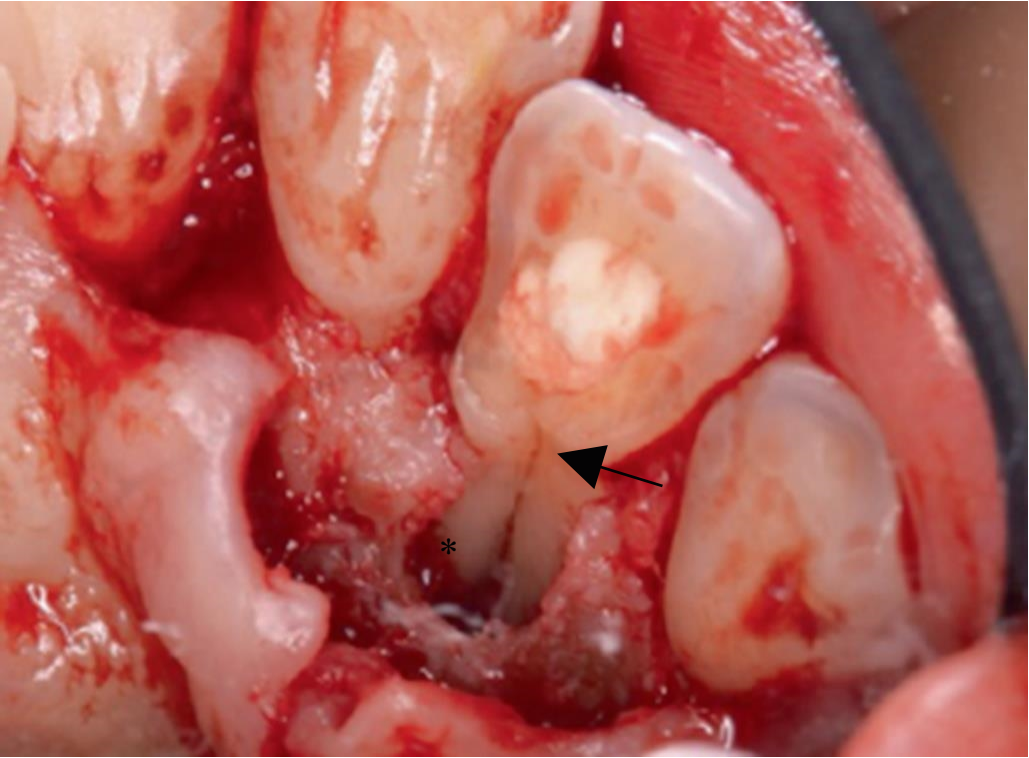
(a)

**Figure:**
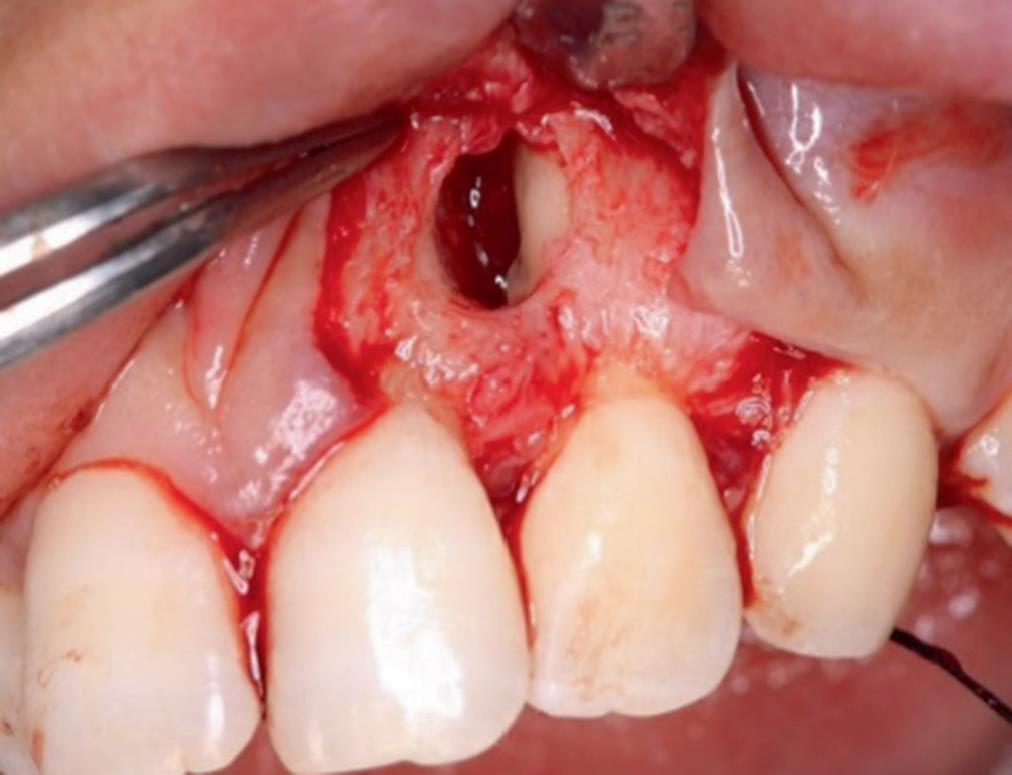
(b)

**Figure:**
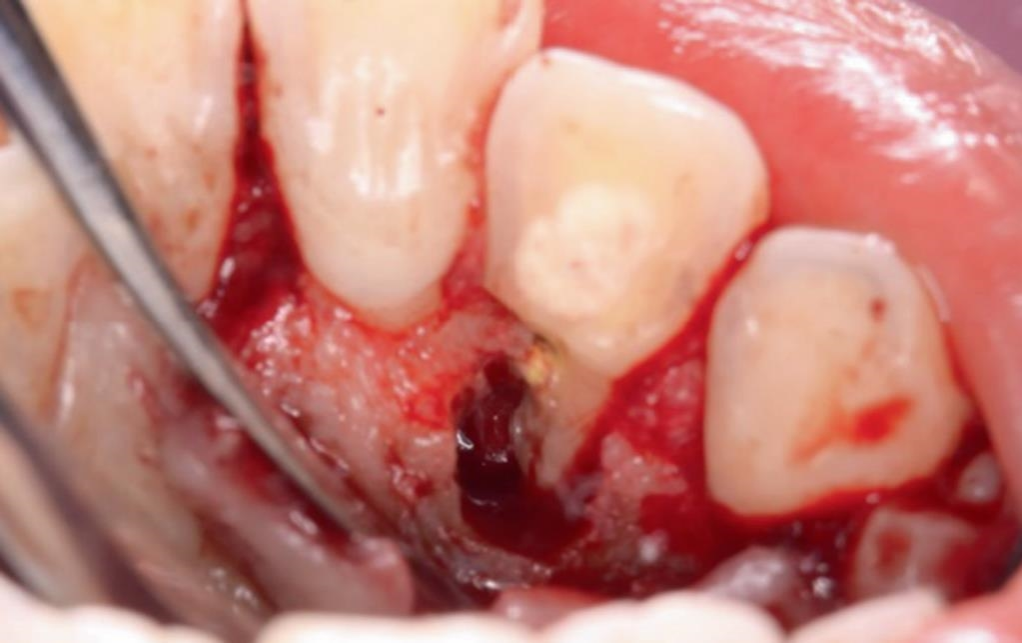
(c)

**Figure:**
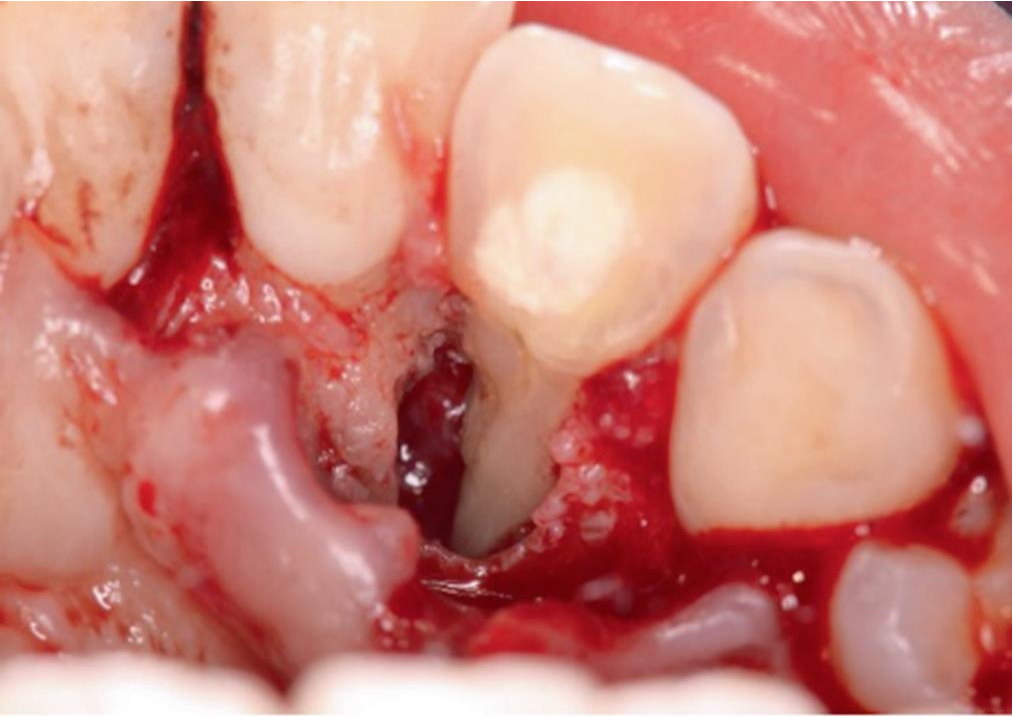
(d)

**Figure:**
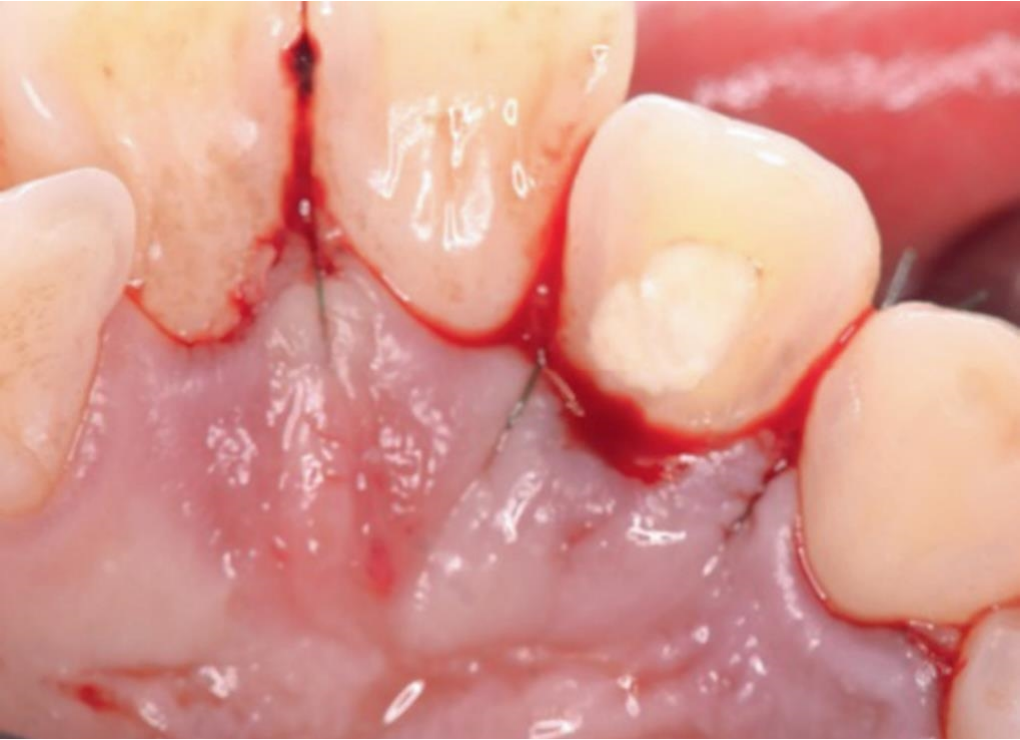
(e)

**Figure:**
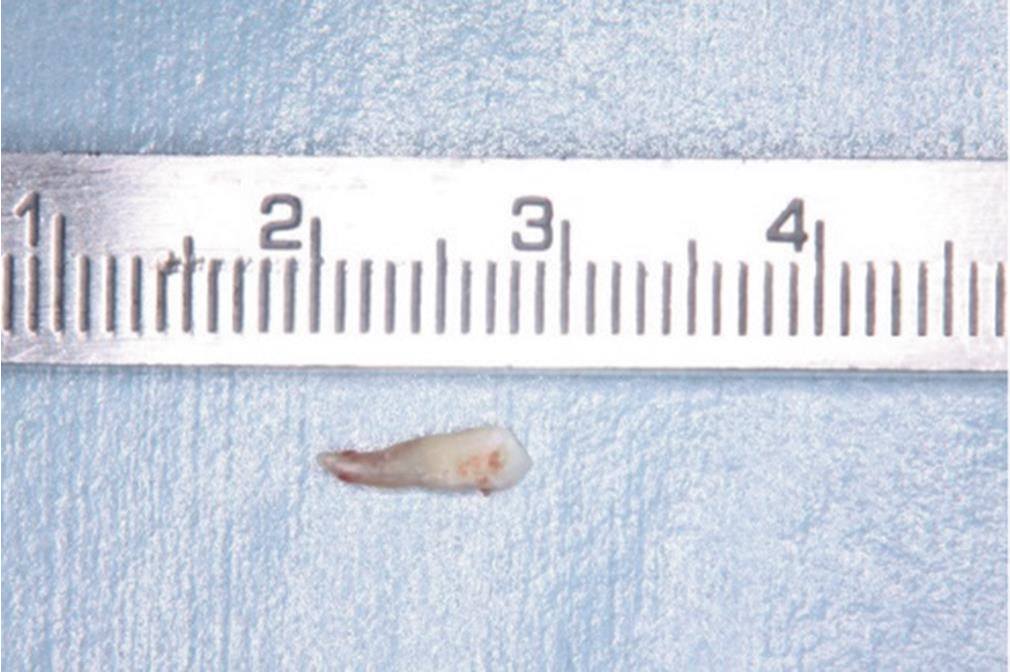
(f)

The access cavity was restored with light‐cured flow composite resin (GRACEFIL Flo) at the next appointment, 1 month after root canal filling.

### 2.5. Treatment Results

**Figure 6:**
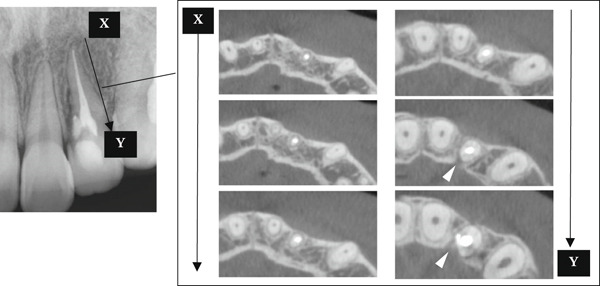
CBCT images of #22 at 24‐month follow‐up. Complete alveolar bone healing is observed except near the palatal cervix (white arrowhead).

Radiographic evaluation at 6 and 24 months showed a gradual increase in radiodensity within the area of the original bony defect, suggesting continued bone regeneration and stabilization of the surrounding periodontal structures. These findings represented clear improvement compared with the immediate postoperative images (Figures 4a, 4b, 4c, and 4d). At the 2‐year follow‐up, there was no evidence of a sinus tract (Figure 5a). The patient was asymptomatic, although a 4‐mm, nonbleeding, periodontal pocket was observed on the palatal aspect (Figure 5b). Progression of hard tissue healing was evident on the CBCT images taken 2 years after the surgery (Figure 6). However, there was insufficient healing of the alveolar bone near the palatal cervix.

Figure 4Postoperative radiographic view. (a) Immediately after surgery. (b) Immediately after root canal filling (1 month after surgery). (c) Six‐month follow‐up. (d) Twenty‐four–month follow‐up.

**Figure:**
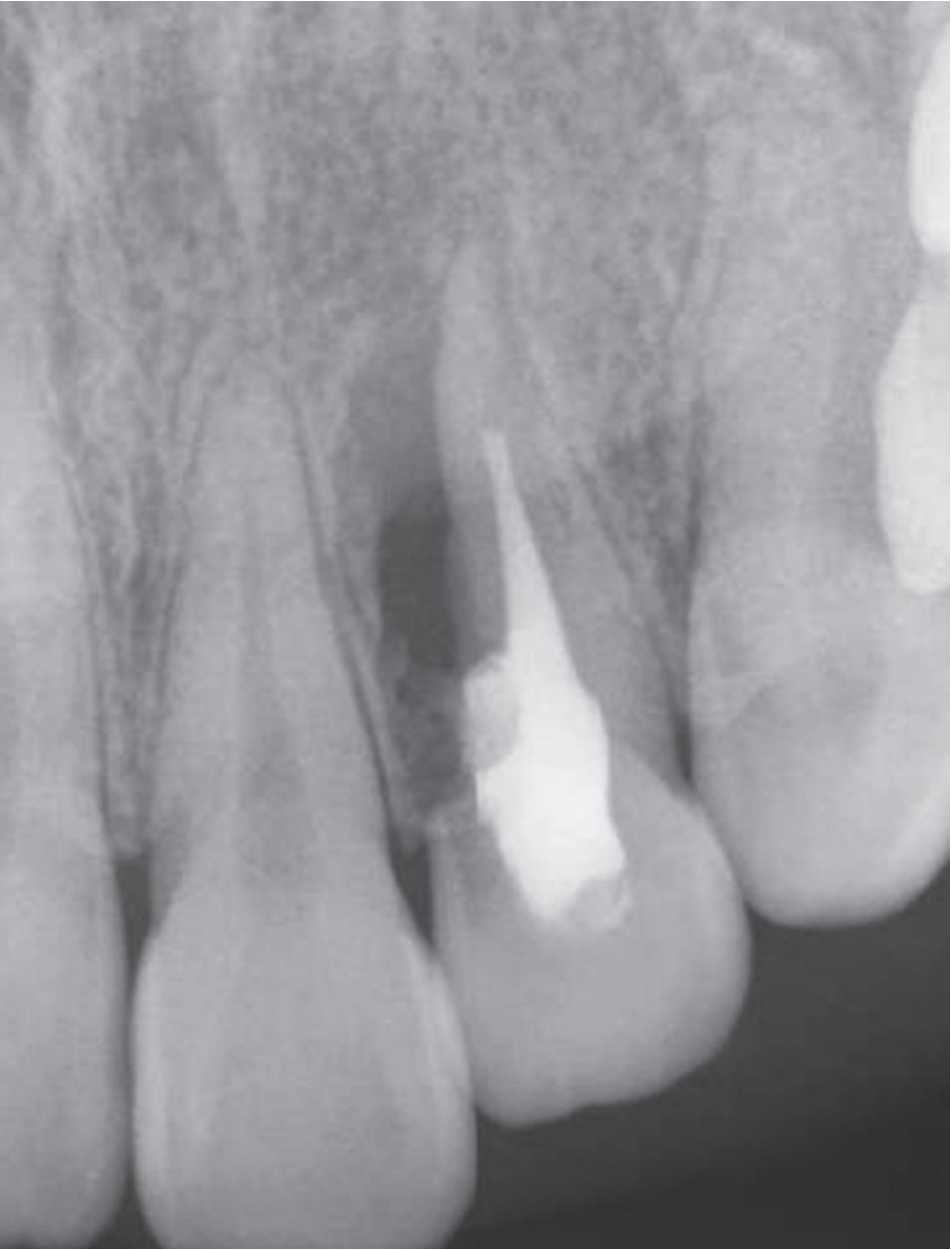
(a)

**Figure:**
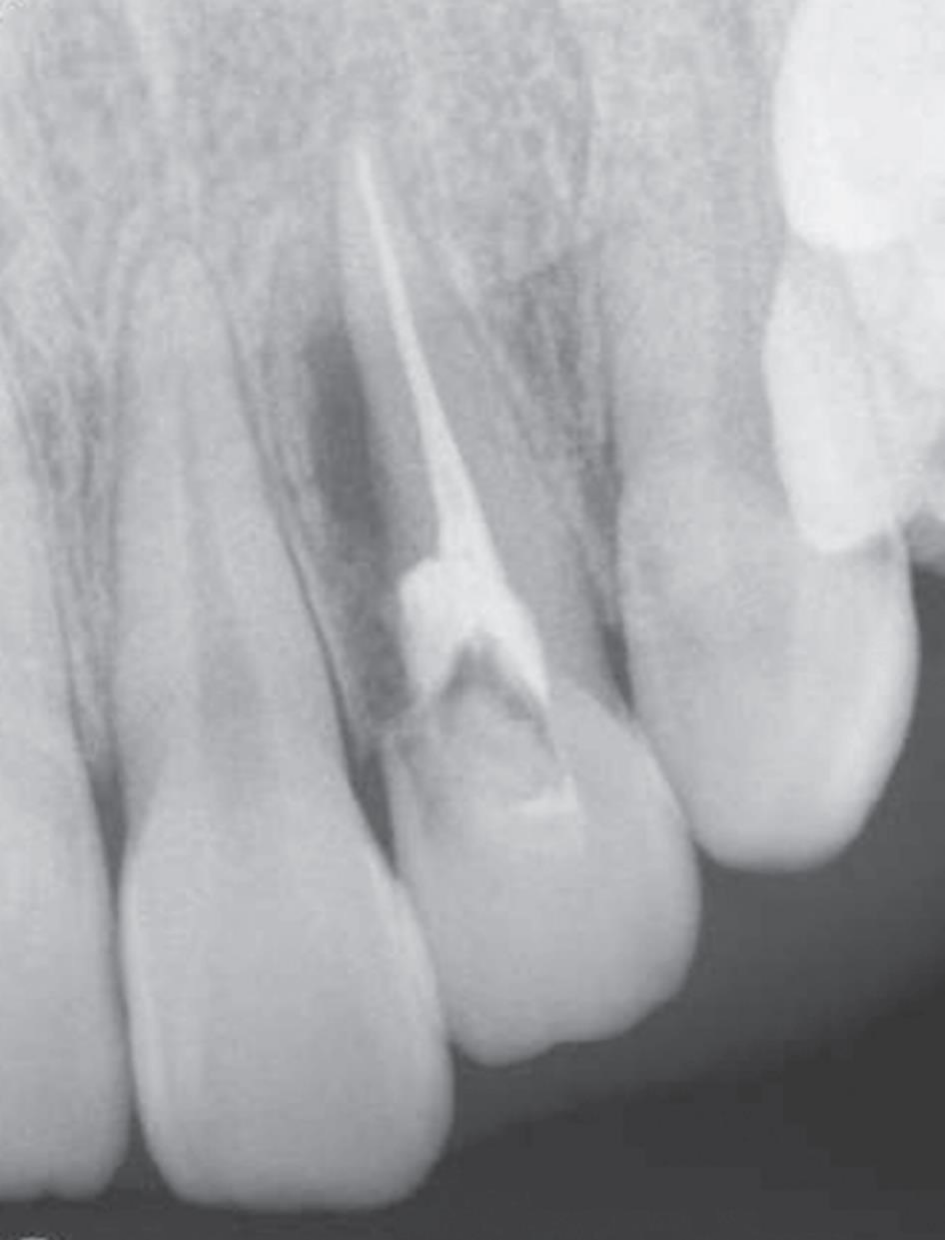
(b)

**Figure:**
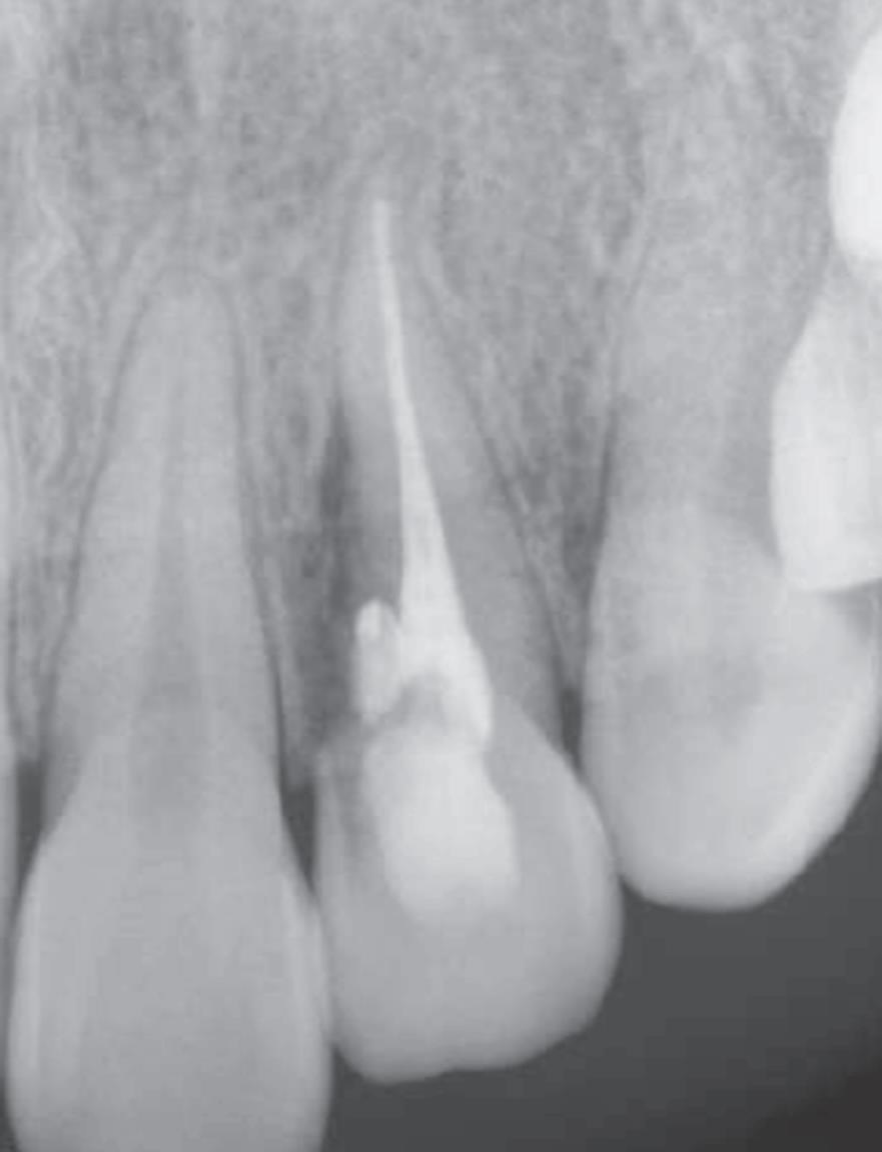
(c)

**Figure:**
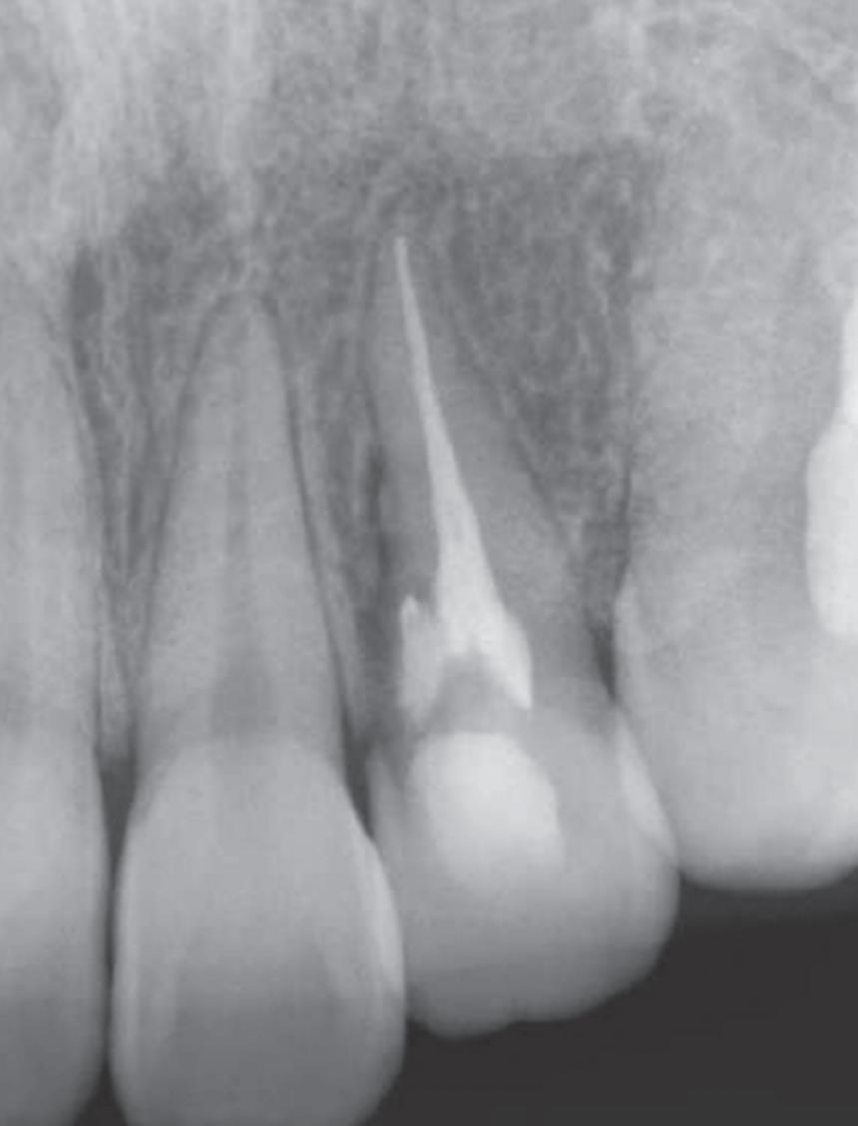
(d)

Figure 5Intraoral photographs at 24‐month follow‐up. (a) There is no evidence of the sinus tract. (b) The 24‐month postoperative photo shows minimal inflammation along with a 4‐mm probing depth.

**Figure:**
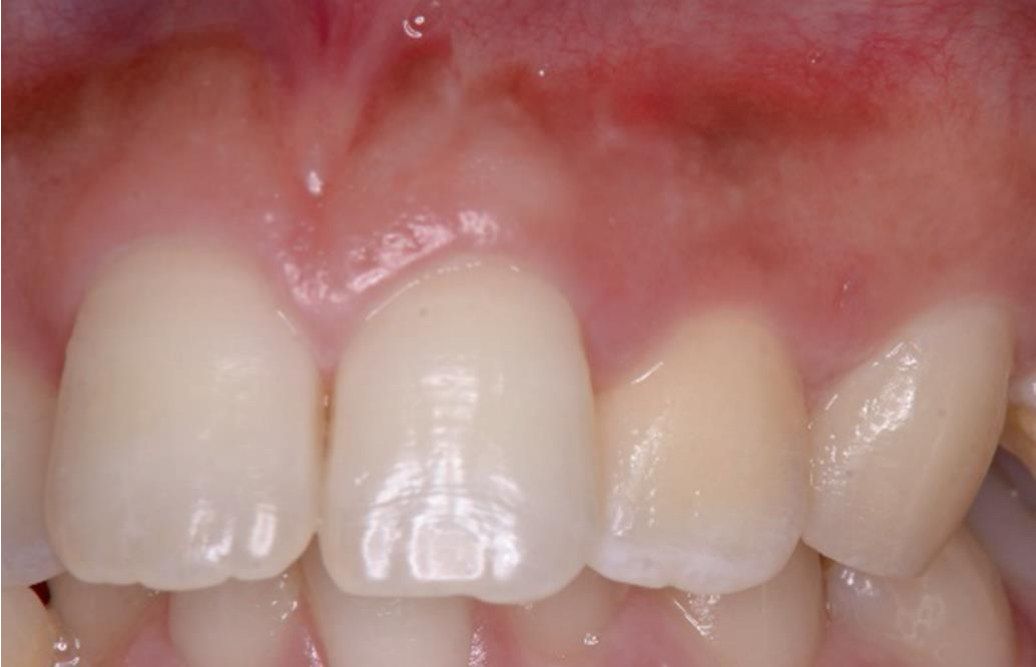
(a)

**Figure:**
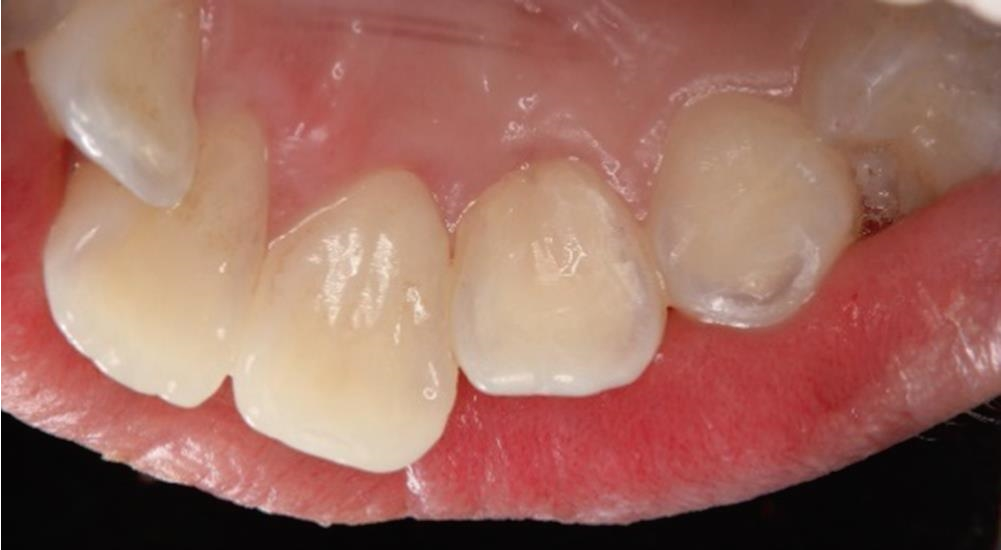
(b)

The patient is pleased with the results, especially because she can now eat comfortably. The follow‐up visits reassured her that the healing was progressing well. Overall, she feels the treatment greatly improved her quality of life.

## 3. Discussion

In the present case, Tooth #22 was classified as a Type III PRG based on preoperative CBCT images. Recently, it has been noted that CBCT images are clinically reliable, readily available, and practical for developing appropriate treatment plans and evaluating prognosis [14]. It is anticipated that the use of CBCT will expand due to its capability to image living organisms with lower radiation exposure than micro‐CT. Gu′s categorization of PRGs into three types is based on micro‐CT imaging of extracted teeth [7]. Therefore, in the future, new PRG classifications may emerge with CBCT.

A PRG can lead to both periodontal and endodontic issues in clinical practice. Therefore, early diagnosis and treatment of PRGs are crucial for improving patients′ prognosis [15]. The fundamental principles for treating PRGs include (i) complete eradication of bacteria, (ii) sealing of the root groove that communicates between the root canal and the periodontal tissue, and (iii) regeneration and healing of periodontal tissue [12]. Shallow grooves are less likely to result in endodontic–periodontal lesions because they obstruct communication between the dental pulp and the periodontal tissue [16]. In cases of mild PRG, which exhibit physiological mobility and shallow grooves, odontoplasty combined with periodontal treatments, such as gingivectomy or subgingival root planning, is recommended [12]. For Type III cases, the groove can be removed using a combination of odontoplasty and sealing materials such as glass ionomer cement or mineral trioxide aggregate (MTA) [17]. In the current Type III case, the accessory foramina of the extra root may have acted as communication channels for periodontal infection and the pulp. Consequently, the extra root was resected and sealed with composite resin.

In the present case, composite resin was selected for sealing the resected root surface after removal of the supernumerary root. Although materials such as glass ionomer cement and MTA are commonly recommended for managing PRGs, each has inherent limitations. Glass ionomer cement offers chemical adhesion and fluoride release, but its comparatively low mechanical strength may compromise long‐term stability in cervical areas subject to functional loading [18]. MTA provides excellent biocompatibility and sealing ability; however, its long setting time and sensitivity to moisture make it less suitable in surgical fields where maintaining a completely dry environment is challenging [19]. In this case, bleeding was adequately controlled during surgery, allowing a sufficiently dry operative field for predictable bonding procedures. This permitted the use of composite resin, which provides immediate polymerization, superior handling, and the ability to precisely contour the resected root surface under direct visualization. These characteristics made composite resin the most appropriate and predictable material for achieving a smooth, well‐adapted surface in this clinical scenario.

In the present case, healing of the alveolar bone near the palatal cervical region was insufficient. Previous studies have shown that a deficiency in marginal bone significantly affects healing outcomes [20]. The complete loss of marginal bone on the palatal side in the present case likely contributed to the difficulty in achieving adequate healing. In addition, a bony fenestration was present on the labial surface of the cortical bone. Taschieri and Pecora reported that the use of GTR (guided tissue regeneration) membrane is effective for lesions larger than 10 mm in diameter and for through‐and‐through defects [21, 22]. In conjunction with the membrane, bone graft materials have been used to regenerate bone defects associated with PRGs [23, 24]. It has been reported that platelet‐rich fibrin application, along with bone grafting, enhanced bone formation in the treatment of PRGs [25]. In this case, 2 years postoperatively, the periodontal pocket at the surgical site measures 4 mm, but no inflammatory findings, such as bleeding, are observed. Therefore, the failure to achieve alveolar bone regeneration may not be due to persistent infection but rather to an insufficient number of endogenous cells necessary for tissue regeneration. Although off‐label in Japan, in this case, the use of GTR membranes or bone grafting materials in combination with flap surgery should have been considered. When planning the regeneration of bone defects associated with PRGs, it is essential to consider the defect size and shape, including the presence or absence of marginal bone loss.

As discussed above, in the present case, it was particularly challenging to achieve regeneration in defects exceeding 10 mm in diameter, especially when accompanied by complete marginal bone loss and through‐and‐through morphology. Pessotti et al. reported the root canal treatment of a two‐rooted maxillary lateral incisor [26]. However, that case did not involve significant periodontal tissue destruction caused by endodontic–periodontal disease attributable to PRG. The present report describes the treatment of a complex, multidirectional bone defect—comprising a palatal marginal defect, a labial fenestration defect, and a penetrating lesion. One noteworthy aspect of this case is its discussion of anatomical regions that pose particular challenges for periodontal regeneration. Looking ahead, therapies such as photobiomodulation, ozone, and probiotics may offer beneficial adjunctive effects in promoting the healing of extensive defects similar to those observed in this case [27–29].

In recent years, cemental tears and invasive cervical root resorption (ICRR) have attracted increased attention [30–32]. Cemental tears refer to the complete or incomplete separation along the cementodentinal junction on the root surface, which can result in periodontal attachment loss. In contrast, ICRR is characterized by unexpected damage that starts in the cervical region of the tooth. The presence of cemental tears and/or ICRR can complicate the diagnosis and treatment of PRGs. Therefore, clinicians should be well informed about these conditions. Although cemental tears and ICRR were not identified in the present case, these entities were discussed because they represent important differential diagnoses for teeth presenting with localized periodontal destruction and deep, isolated periodontal pockets—clinical features that can also be associated with advanced PRGs. Both conditions may exhibit patterns of attachment loss and bone defects that resemble PRG‐related lesions, and failure to distinguish among them may lead to incomplete or inappropriate treatment. Accordingly, awareness of these conditions is essential when evaluating complex cervical lesions, and clinicians should carefully rule them out when establishing a diagnosis and treatment plan for PRG‐associated periodontal defects.

## 4. Conclusion

Achieving good long‐term outcomes in the treatment of Type III PRGs requires accurate diagnosis using CBCT, resection of an accessory root to remove the groove, debridement of the root surface, and root canal treatment under infection control.

## Author Contributions

Concept—K.T.; supervision—T.N. and H.S.; literature search—Y.T., R.K., and K.Y.; critical review—all authors.

## Funding

The authors declared that this study received no financial support.

## Ethics Statement

This study was conducted in accordance with the Declaration of Helsinki. This case report describes the treatment course of a single patient.

## Consent

Written, informed consent was obtained from the patient for the publication of the case report and the accompanying images.

## Conflicts of Interest

The authors declare no conflicts of interest.

## Data Availability

The data that support the findings of this study are available upon request from the corresponding author.
